# Estimating the Future Impact of a Multi-Pronged Intervention Strategy on Ocular Disease Sequelae Caused by Trachoma: A Modeling Study

**DOI:** 10.3109/09286586.2015.1081249

**Published:** 2015-12-14

**Authors:** Manoj Gambhir, Nicholas C. Grassly, Matthew J. Burton, Anthony W. Solomon, Hugh R. Taylor, David C. Mabey, Isobel M. Blake, María-Gloria Basáñez

**Affiliations:** ^a^ Department of Epidemiology and Preventive Medicine, Monash University, Melbourne, Victoria, Australia; ^b^ Department of Infectious Disease Epidemiology, Imperial College London, London, UK; ^c^ MRC Centre for Outbreak Analysis and Modelling, Department of Infectious Disease Epidemiology, Imperial College London, London, UK; ^d^ Clinical Research Department, Faculty of Infectious and Tropical Diseases, London School of Hygiene & Tropical Medicine, London, UK; ^e^ Indigenous Eye Health Unit, Melbourne School of Population Health, The University of Melbourne, East Melbourne, Victoria, Australia

**Keywords:** Blindness, *Chlamydia trachomatis*, elimination program, mathematical modeling, ocular sequelae

## Abstract

*Purpose*: Trachoma control programs are underway in endemic regions worldwide. They are based on the SAFE strategy (Surgery for trichiasis, Antibiotic distribution, Facial cleanliness, and Environmental improvement). Although much is known about the effect of community-wide treatment with antibiotics on the prevalence of *Chlamydia trachomatis*, the impact of the SAFE strategy on severe ocular disease sequelae (the main focus of the Global Elimination of blinding Trachoma by 2020 program) remains largely unknown.

*Methods*: We use a mathematical model to explore the impact of each of the components of the SAFE strategy, individually and together, on disease sequelae, arising from repeat infection and subsequent conjunctival scarring. We ask whether two elimination goals, to reduce the prevalence of trachomatous trichiasis to 1 per 1000 persons, and the incidence of corneal opacity to 1 per 10,000 persons per annum, are achievable, and which combinations of interventions have the greatest impact on these indicators.

*Results*: In high prevalence communities (here, >20% infection of children aged 1–9 years), a combination of efforts is needed to bring down sustainably the prevalence and incidence of ocular disease sequelae.

*Conclusion*: The mass delivery of antibiotics is highly beneficial for the clearance of infection, inflammation and prevention of subsequent scarring, but needs to be supplemented with sustained reductions in transmission and surgery to consider realistically the elimination of blindness by the year 2020.

## Introduction

Trachoma remains the world’s leading infectious cause of blindness. In 2003, the World Health Organization (WHO) estimated that 84 million people had active disease (an inflammatory conjunctivitis) that could lead to visual impairment, and that 7.6 million were severely visually impaired or blind as a result of trachoma.^1^ Recently revised estimates have halved the number of people with active trachoma, probably due to the collection of more accurate data since the introduction of the SAFE strategy (Surgery for trichiasis, Antibiotic treatment of the causative bacteria *Chlamydia trachomatis*, Facial cleanliness, and Environmental improvement),^2^ the success of SAFE implementation, and socioeconomic improvements in endemic areas.^3^ Yet, the number of people with trichiasis (inward turning of eyelashes causing corneal damage that will ultimately lead to blindness) is currently about 8.2 million.^3^

The WHO’s simplified grading scheme^4^ discriminates follicular and inflammatory trachoma (TF and TI, respectively). With repeated ocular chlamydial infection, episodes of inflammation lead to a scarring immunopathological response, defined as trachomatous scarring (TS).^4^ Further inflammation leads to worsening of scarring that may turn the eyelid inward and cause eyelashes to abrade the corneal surface (trachomatous trichiasis, TT). If trichiasis is not treated through surgical intervention, the eye may become irreversibly damaged leading to corneal opacity (CO) and eventually blindness.

Mass drug administration (MDA) programs, using the antibiotic azithromycin, are part of the SAFE strategy (the “A” component). The International Trachoma Initiative (ITI) estimates that by 2015 MDA programs will be active in 42 countries.^5^ Recently, the London Declaration on Neglected Tropical Diseases (endorsed on 30 January 2012), pledged to support efforts to help eliminate blinding trachoma by the year 2020.^6^ Because the time between the administration of treatment and its observed effects on disease (and, in particular, trichiasis and CO) can be very long, mathematical models can provide valuable tools for predicting the long-term effects of treatment and for assessing the feasibility of the 2020 goals. In previous papers,^7^ we have shown that relatively simple mathematical models reproduce adequately many of the qualitative and quantitative patterns of infection and disease observed in trachoma-endemic communities prior to the introduction of MDA.

This paper investigates the effects of perturbing the model through simulated treatment schedules to ascertain (1) what effect these control interventions might have on ocular disease, (2) whether the Global Elimination of blinding Trachoma by 2020 (GET2020) program goals are achievable, and (3) how long these goals might take to achieve.

## Materials and Methods

Since the goals set by the WHO GET2020 alliance primarily concern the elimination of prevalent cases of TT (and, formerly, incident cases of CO), as well as a reduction in the prevalence of active trachoma (TF, TI),^8^ it was necessary to construct a mathematical model that explicitly included the progress of individuals to such disease sequelae.

The model presented by Gambhir and co-authors,^7^ fitted to age-stratified prevalence data of TS and TT for the high prevalence setting of Kongwa, Tanzania, described by West and colleagues,^9^ was used as the basis from which to investigate the treatment schedules explored in this paper. Details of the model structure and the values of the parameters are given in the Supplemental Appendix (available online only). The model used here is similar in structure to that published by Lietman and co-workers,^10^ and is based on the simple susceptible-infected-susceptible framework; here, however, the infection transmission mechanism is simplified to allow for an additional model feature, namely the path individuals take to the development of disease sequelae. We considered a treatment naive high-prevalence setting to be a reliable source of data for the prevalence of disease sequelae since in such a setting the influence of environmental or socioeconomic secular trends will have been minimal, and it is reasonable to assume that infection dynamics were at equilibrium, allowing us to clearly associate infection transmission with disease sequelae.

Using estimated transmission parameters from this high prevalence setting (>20% infection of children aged 1–9 years), we simulated the administration of multiple antibiotic annual treatment rounds to populations whose initial infection prevalence level was high. We examined the impact of MDA alone, and a combination of surgery, MDA and “transmission reduction,” on each of the disease sequelae prevalence levels, with host age and time, and the impact of these interventions on the incidence of severe disease sequelae. We refer to the third of the interventions we tested as “transmission reduction” rather than F&E since the effectiveness of F&E is not well understood and we wanted to prevent readers from conflating our simulated intervention with F&E. It is likely that F&E (and, indeed, Water, Sanitation and Hygiene; WASH) interventions would be modeled as “transmission reduction,” but the disease modeling community awaits further evidence before making this link more explicit.

### Model and Assumptions

**FIGURE 1.**
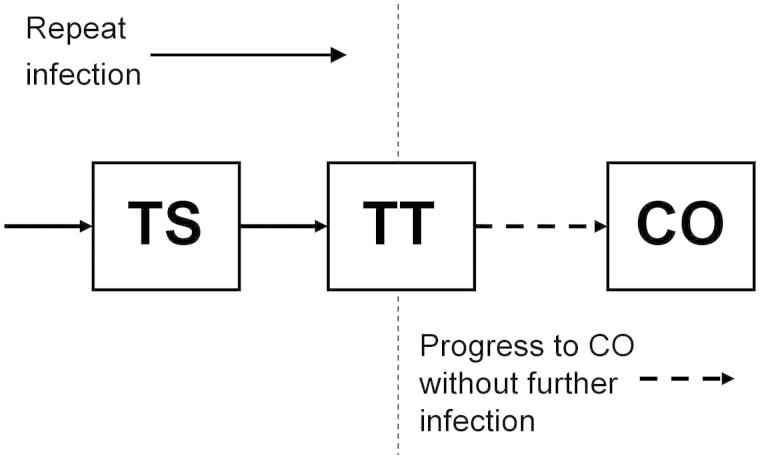
Trachoma disease model diagram. Compartmental disease model based on the ladder of infection framework of Gambhir and colleagues^7^ with an extension to take into account development of corneal opacities (CO) from the population of those who previously developed trachomatous scarring (TS) and then trichiasis (TT). The disease states TS and TT are developed through repeat infection, but development of CO is not linked to further infection and occurs at a constant rate as a consequence of mechanical corneal damage incurred by those with TT (see Supplemental Appendix for model parameters).

The mathematical framework of our model has previously been described in detail,^7^ its prototype having been presented by Gambhir and co-authors. Briefly, the model is based on the process of ocular reinfection of individuals with *C. trachomatis* as they come into contact with others who are infected. Progressive conjunctival fibrosis/scarring (TS) is believed to be initially caused by recurrent *C. trachomatis* infection. However in the late stages of the disease, other stimuli such as non-chlamydial bacterial infection, may also contribute to this inflammatory-cicatricial process.^11^ Members of the population eventually accumulate threshold numbers of infection episodes, and these thresholds are interpreted as points beyond which individuals show signs of the disease sequelae TS and TT. The third, and more severe disease sequela, CO, has a different etiology, which we model by assuming that once individuals show signs of TT, mechanical damage of the cornea will gradually cause them to progress to CO, regardless of whether they continue to be infected (Figure 1).

The rate of recovery of individuals from an episode of infection increases with age,^12^ and the model includes this more rapid recovery by postulating its cause as the increasingly rapid clearance of each successive infection.^13^ Additionally, the infectivity of individuals is assumed to be proportional to the bacterial infection load they carry and this value drops with age, which is likely to be another consequence of acquired immunity.^14^^,^ 15 Model populations were parameterized by fitting the model to endemic infection and disease sequelae prevalence data from Kongwa, Tanzania. This model was also found to exhibit disease sequelae incidence rates that closely matched those published for this district.^16^ Thus calibrated to the disease sequelae “steady-state,” we had a model that could be used to simulate control interventions. Mathematical details of the model are presented in the Supplemental Appendix.

### Simulation of the Interventions Individually and Together

We first ran the model for a 200-year equilibration period to ensure a steady state was reached. We simulated control scenarios based on the transmission and disease parameters estimated from fitting the model to a high prevalence setting at baseline (Kongwa district, Tanzania^7^^,^ 9). Each intervention component of the full strategy was simulated at a high, medium or low intensity of effort (as detailed in Table 1). Antibiotic treatment (A), was simulated by shifting a proportion of the population in the infected classes to the closest susceptible classes according to an antibiotic efficacy of 95%,^17^ and a therapeutic coverage level equal to 86% of the total population, which was the level achieved by the first MDA round in the high prevalence field study in Kongwa.^9^ At the time of treatment, a population fraction given by *efficacy × coverage* had its bacterial carriage effectively reduced to zero. Each MDA round was conducted in successive years (and each set of three MDA rounds was conducted in successive 3-year periods). The simulation results were examined to determine the effect of antibiotic treatment on age-dependent prevalence and incidence levels of disease sequelae over the whole population. The “transmission reduction” intervention was simulated as an annual linear decrease in the transmission rate over such a period that the final transmission rate was 10% of its initial value (i.e. a high-endemicity setting had been transformed into a low-endemicity one). The rates of decrease were chosen so as to achieve this decline in transmission in 10, 20 or 30 years. The slowest-acting of the “transmission reduction” interventions might be interpreted as the rate associated with steady economic development and the resulting environmental improvement, an example of which occurred in the Gambia between the mid-1980s and the mid-1990s without concerted intervention.^18^ The provision of surgery (S) for TT was simulated as a decrease in the number of cases of TT over time at a rate such that, from the onset of community surgery, the average amount of time an individual spends with TT would be 2, 5 or 10 years (ie, mean residence time in the TT disease state is 2, 5 or 10 years, the mid value of which is in line with current WHO guidelines^1^). Note that, while the population to whom surgery is provided no longer suffer from TT, they are still subject to the rest of the infection and recovery dynamics in the model. So, transmission of infection across the population is not affected by surgery. When these three interventions were implemented together (in pairs, or all three at once), the intensities of the interventions were combined like-with-like, so that interventions were implemented at a low level for all three component interventions (i.e. low S + low A + low “transmission reduction”). All seven possible intervention combinations are detailed in Table 2. The time taken to achieve the elimination or control goals was estimated by focusing on the two goals relating to the elimination of blinding trachoma; (1) reduction in the prevalence of TT to ≤1 case per 1000 persons, and (2) reduction in the number of incident cases of CO to <1 per 10,000 persons per annum,^8^^,^ 19 a previously codified goal for elimination, although this is now no longer a formal target due to the difficulty in estimating CO incidence in an endemic area. Life table estimates from the WHO for the year 2001 were used in the model for the age-specific death rates and the overall population birth rate for Tanzania.^20^

**TABLE 1.** Trachoma treatment schedules implemented at three individual control interventions and three intensity levels. Each set of three simulated annual MDA rounds, for medium and high intensities of the antibiotic intervention, are conducted immediately following the previous three annual rounds.

		Intensity of control effort		
Intervention	Definition of intervention	Low	Medium	High
Surgery	TT case backlog clearance time	10 years	5 years	2 years
Antibiotics	Annual MDA rounds at mean 86% coverage	3 (3 rounds followed by population prevalence review is recommended by WHO)	3 + 3 annual rounds (also at mean 86% annual coverage)	3 + 3 + 3 annual rounds (also at mean 86% annual coverage)
Transmission reduction	Reduction of transmission rate per year (until final transmission rate is 1/10 of its initial value)	3%	4.5%	9%

MDA, mass drug administration; TT, trachomatous trichiasis; WHO, World Health Organization.

**TABLE 2.** Global Elimination of blinding Trachoma by 2020 (GET2020) program goals and the implementation of components of the full intervention strategy individually and in concert. Each component of the full intervention strategy is implemented at a low, medium or high intensity, corresponding to the schedules detailed in Table 1. Outcome measures are stated here such that a value of 1.0 represents achievement of the GET2020 goals. The dark-shaded cells are those in which the GET2020 goals have been achieved and cells that are light-shaded correspond to schedules in which the goal is almost achieved (i.e. within 100% of the goal).

		Prevalence of TT post-intervention (cases per 1000 persons)			Incidence of CO post-intervention (cases per 10,000 persons per annum)		
Intervention	Intensity of control effort	5 years	10 years	20 years	5 years	10 years	20 years
S	Low	9.4	7.4	6.3	3.3	2.6	2.1
	Medium	3.9	3.9	3.9	1.3	1.3	1.3
	High	1.8	1.8	1.8	0.6	0.6	0.6
A	Low	13.5	13.6	13.6	4.6	4.6	4.6
	Medium	12.8	13.0	13.1	4.3	4.4	4.4
	High	12.8	12.3	12.5	4.3	4.2	4.2
TR	Low	13.6	12.6	9.1	4.6	4.3	3.1
	Medium	13.4	11.9	6.8	4.6	4.1	2.4
	High	12.8	9.7	4.5	4.4	3.4	1.6
S + A	Low	9.1	7.2	6.2	3.2	2.5	2.1
	Medium	3.1	3.6	3.6	1.1	1.2	1.2
	High	1.4	1.6	1.7	0.5	0.5	0.6
S + TR	Low	9.1	6.4	3.1	3.2	2.2	1.1
	Medium	3.4	2.6	0.6	1.2	0.9	0.2
	High	0.6	0.3	0.2	0.4	0.1	0.1
A + TR	Low	13.6	12.6	9.1	4.6	4.3	3.1
	Medium	13.4	11.6	6.2	4.6	4.0	2.2
	High	12.8	9.1	4.1	4.4	3.2	1.4
S + A + TR	Low	9.1	6.4	3.1	3.2	2.2	1.1
	Medium	3.4	1.2	0.5	1.2	0.9	0.2
	Hi	1.2	0.1	0.1	0.4	0.1	0.1
None		14.0	14.0	14.0	4.7	4.7	4.7

S, surgery; A, antibiotics; TR, transmission reduction; TT, trachomatous trichiasis; CO, corneal opacities.

Table S1 from the Supplemental Appendix summarizes the variables and parameters of the model, provides their values when these were chosen from previous work or set to mimic different endemicity levels, and indicates which parameters were estimated by fitting the model to the data.

We also calculated the time course of the prevalence of TS, TT and CO and the incidence of CO for each of the interventions. Each of the interventions was implemented in populations that were initially in a high prevalence state for trachoma infection (>20% infection prevalence in children 1–9 years^21^). The reason that we did not investigate the effect of these interventions on sequelae levels in medium- and low-endemicity settings (10–20%, or <10% infection prevalence, respectively^21^) is because, in a steady state, the infection process is not sufficient to generate new cases of TT and CO in these lower endemic settings. However, it does appear to be the case that several currently low-endemicity regions were initially high-endemicity and, therefore, a higher prevalence than expected of disease sequelae may be found in these settings. Our intervention simulations mimic this effect by considering “transmission reduction” interventions on the high-endemicity population, which rapidly reduce infection but leave previously developed TS, TT and CO, and allow for the continuous generation of CO cases, which do not require further infection.

## Results

### Prevalence of Disease Sequelae Following Implementation of the Components of the Full Intervention Strategy Individually and in Combination

**FIGURE 2.**
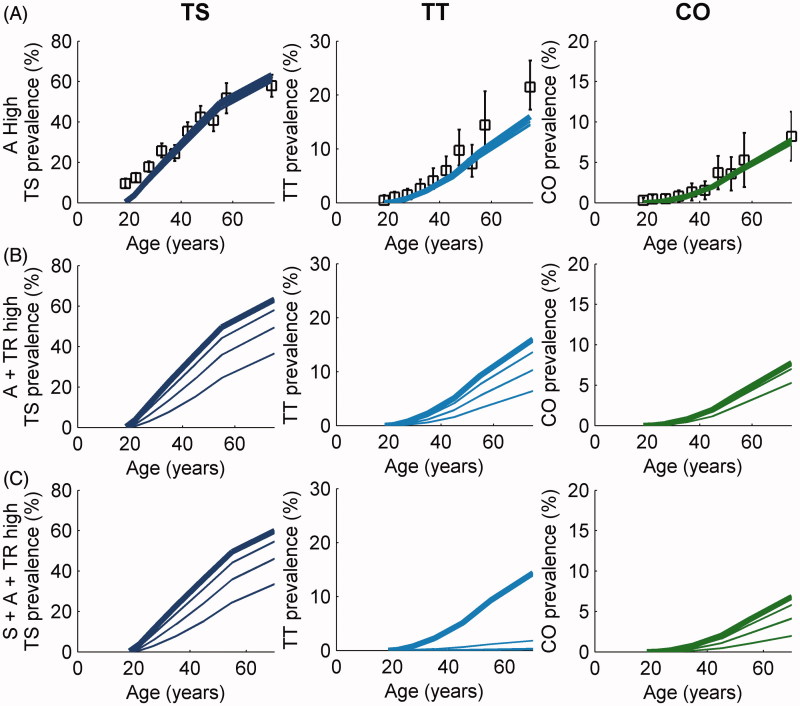
Response of trachoma disease sequelae prevalence to treatment. Simulated age-dependent prevalence of disease sequelae (trachomatous scarring [TS], dark blue lines [left]; trachomatous trichiasis [TT], light blue lines [middle]; and corneal opacities [CO], green lines [right]), in a high-endemicity trachoma setting at baseline, following three treatment schedules; (A) antibiotics (A) only, at high intensity; (B) A+ transmission reduction (TR) both at high intensity; (C) surgery (S) + A + TR all at high intensity. In each plot, the thicker lines represent baseline prevalence over age and each subsequent lower curve is plotted at 5-year intervals such that the bottom-most curves represent the prevalence levels 20 years after baseline. In (A), the markers indicate the prevalence values of TS, TT, and CO observed in Kongwa, Tanzania^9^ with error bars representing 95% confidence intervals of the collected dataset.

Figure 2 illustrates the impact on the age-dependent prevalence of disease sequelae of the three interventions selected from the full set described in Tables 1 and 2. The first of these (Figure 2A) shows that the impact on prevalence of sustained antibiotic intervention alone, acting at a high intensity of control effort (three annual rounds of MDA followed by a further two sets of three annual rounds) is such that there is little change in prevalence; the transmission rate is too high and infection rebounds very rapidly following MDA, which in turn sustains a steady number of new cases of TS, TT and CO.

The combination of antibiotic MDA and “transmission reduction” (Figure 2B), each at a high level of intensity, reduces the transmission rate to 10% of its initial value in 10 years and effectively transforms the transmission setting from a level of high- to low-endemicity. However, the impact of this intervention takes years to become evident in the prevalence of scarring sequelae, and it is only 20 years after the cessation of the intervention that significant changes in the prevalence pattern are seen. However, when the entire strategy is deployed (Figure 2C), the prevalence of TT can be reduced to 0% in 20 years because surgery for trichiasis is also intensely implemented. The incidence of CO is also more rapidly reduced by the full strategy, but there is a delay in its reduction due to the slow disappearance (via any-cause mortality) of those cases of CO that arose prior to the intervention. Other than the surgery-induced large decline in TT prevalence shown in Figure 2C, the declines seen in sequelae prevalence levels are largely demographic; they occur as a consequence of natural mortality in populations with TS, TT and CO, since surgery is the only effective measure available to reverse progress toward more severe disease among individuals with TT.

### Prevalence and Incidence of Disease Sequelae on Implementation of Interventions

**FIGURE 3.**
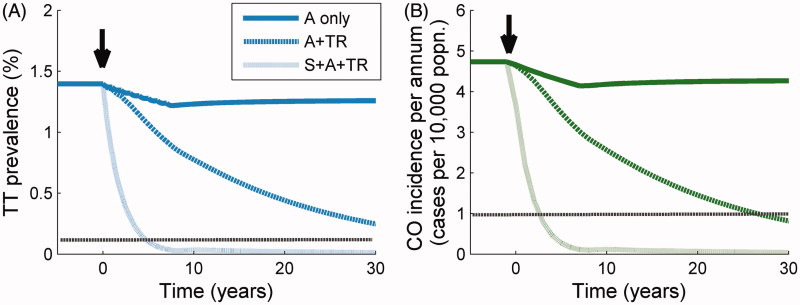
Response of trachomatous trichiasis (TT) prevalence and corneal opacity (CO) incidence to treatment. Trend in: (A) prevalence of TT, and (B) incidence of CO through time following three treatment schedules initiated at 0 years (black arrow). From the uppermost to the bottom curve, the population is subjected to mass drug administration (MDA) at high intensity (dark solid line), MDA + transmission reduction (TR) both at high intensity (upper dark dotted line), and MDA + TR + surgery (S), all at high intensity (lower light dotted line). The horizontal dotted line represents the relevant threshold for the Global Elimination of blinding Trachoma by 2020 (GET2020) program goals (a level of one case of CO per 10,000 persons per annum, and a prevalence of one case of TT per 1000 persons).

Figure 3 illustrates the population prevalence of TT and incidence of CO over time during and following the three selected treatment schedules that we used to project the prevalence profiles in Figure 2. The small drop in these measures for the “antibiotic only” schedule (solid lines, Figure 3A and B), in which nine annual MDA rounds are administered, creates a small but long-lasting decrease in both disease levels due to the decline in the number of new cases generated through infection, as well as the damage due to TT. This benefit of antibiotic administration should not be underemphasized since it will result in fewer cases of blindness and severe discomfort, but there will be a steady tendency to rebound (to pre-intervention levels) of both disease sequelae. The time course of the incidence of CO and the prevalence of TT following the initiation of the A + “transmission reduction” intervention (dashed line, Figure 3A and B) is much more persistently downward, but it is not until surgery is added to the combination of interventions (dot-dashed line, Figure 3A and B) that the prevalence of TT and incidence of CO is brought down sufficiently quickly to make achieving the GET2020 program goals by the year 2020 feasible.

### Incidence of CO, Prevalence of TT and the GET2020 Program Goals

Results from a comprehensive exploration of the impact of each of the components of the full intervention strategy, individually and together, are given in Table 2. The outcome measures included in the table are those associated with the GET2020 program goals, namely the incidence of CO (persons per 10,000 per annum), and the prevalence of TT (as a percentage) 5, 10 and 20 years following the beginning of the interventions. Here, it can clearly be seen which control strategies employed alone or together result in the achievement of the GET2020 program goals. The strategy components and combinations that succeed are dark-shaded and those that bring sequelae levels very close to the goal (here quantified as a CO incidence of two persons per 10,000 per annum, or TT prevalence of two persons per 1000) are light-shaded. Several general trends can be seen; first, the inclusion of greater numbers of the intervention components leads more readily to the accomplishment of the (current or former) GET2020 program goals; second, the implementation of surgery is immensely important and, indeed, surgery is the intervention component that most surely results in the achievement of the sequelae-related goals when implemented *alone*; third, the TT goal is not quite as readily achieved as the CO goal, which is a consequence of the time delay in the drop in prevalence compared with incidence, resulting in the achievement of this goal taking longer than the year 2020 target.

## Discussion

In this paper, we investigated the impact on TS and blinding disease sequelae of the components of a multi-pronged intervention strategy implemented at various degrees of control effort intensity, individually and together. In the absence of data on the long-term impact of treatment on these sequelae, mathematical models may provide helpful projections of treatment impact to help assess the feasibility of reaching the year 2020 goals.

Referring to the three questions we sought to answer at the beginning of this article, we find that: (1) Increased intensity of all interventions will more quickly positively impact ocular disease incidence and prevalence levels. Increased levels of surgery will most surely impact the indictors included in the GET2020 program goals. However, surgery alone is a stopgap measure, until transmission can be reduced and, once reduced, the impact on ocular disease of MDA is also enhanced. (2) The GET2020 program goals are still achievable in theory if all interventions are implemented simultaneously (and with the proviso that our “transmission reduction” intervention may not be easily implemented on the ground). (3) Under current intervention regimes and in high-transmission settings (i.e. in hyperendemic communities) the GET2020 program goals may be difficult to achieve sooner than around 20 years from now.

A few studies have observed declines in prevalence of infection and disease that have occurred either disproportionately with respect to the antibiotic treatment administered,^22^^,^ 23 or even entirely without antibiotics.^24^ The elimination of ocular chlamydial infection from many countries in which it was previously endemic preceded the widespread use of antibiotic treatment for trachoma and was probably related to economic and infrastructural improvement.^25^ The facial cleanliness (F) and environmental improvement (E) components of the SAFE strategy seek to lower the level of bacterial transmission so that antibiotic treatment has a greater impact, or the infection is driven to elimination without the need for antibiotics. We have shown that in high-endemicity settings, infection may rebound too quickly to give rise to a sustained reduction of the incidence of disease sequelae when antibiotic treatment is administered alone. In such cases, the S, F and E components of the SAFE strategy would appear to be crucial and could result in the local elimination of infection as well as a rapid reduction in the number of new cases of CO and blindness.^25^ Our model does not explicitly include the F&E intervention, since little is known of its effectiveness in reducing population infection and disease. However, a recent meta-analysis of the available data for WASH interventions and their preventative impact on trachoma has found that a clean face is highly associated across many studies with the absence of TF.^26^ Quantitative estimates of risk reductions such as these may be translated into changes in model parameters in future work that explicitly examines the F&E intervention. Background secular trends (e.g. non-programmatic environmental improvements, washing practices, and fly density reduction) would be similarly modeled.

The main GET2020 program goal, namely, the elimination of incident blindness as a result of trachoma by 2020,^1^^,^ 8 ^,^ 19 is linked, in the model, to the interruption of the transmission of infection and the slowing down or stopping of progress along the ladder of infection, although we represent the path from TT to CO as a process with constant rate without the need for further infection. We find that the incidence of CO drops by around 20% of its initial value following nine annual MDA rounds (Figure 3B), and it is only after the transmission rate has been reduced very substantially and surgery for TT is intensely provided that these sequelae are no longer incident.

### Model Limitations

The model implemented here is limited by its simplicity. First, it is deterministic, which precludes the possibility of investigating infection elimination, second, it assumes that contact between individuals is homogeneous between sexes, although not over ages, third, it does not take into account differences in transmission within and between households, a heterogeneity in transmission that has been shown to be important in trachoma epidemiology,^27^ an issue investigated in a recent modeling study,^28^ and fourth, the “transmission reduction” intervention was modeled as a linear decline in transmission and is not currently calibrated to published effectiveness levels, which remain very uncertain and are yet to be evaluated in highly-controlled study settings. This uncertainty is very important, since estimates of the time to achieve elimination under “transmission reduction” are strongly dependent on its effectiveness. We have accounted for this by performing time-to-elimination calculations over a range of “transmission reduction” effectiveness values, to allow readers to gain insight into the change in likelihood of elimination with increases in intensity of each intervention. The simplicity of the current model therefore limits investigations into the effects of targeted treatment (e.g. to children, or to households in which there is at least one case of active disease). Our simplified representation of trachoma natural history and, particularly, the abrupt threshold number of prior infections necessary to reach the disease states TS and TT, might also be modified in future work by associating a probability with the development of TS and TT over a range of infection levels, or by using a disease sequelae model closer to those used for helminth-induced disease, in which the population moves towards severe disease states, with a rate depending on the number of prior infections.^29^ Fifth, the observed persistence of active disease following the elimination of infection^11^^,^ 30 underscores the idea that repeat infection does not drive disease alone, and forecasting efforts will need to account for this. Finally, our results were obtained by fitting our model to data from Kongwa district, Tanzania, and our conclusions would be more robust if, in future work, we used the model fit to other data sets.

### Policy Implications

The policy implications of the tabular summary of all intervention permutations (Table 2) are clear; for communities with high infection prevalence at baseline, a combination of efforts is needed to bring down the prevalence and incidence of sequelae sustainably to achieve the GET2020 program goals. Table 2 highlights the importance of surgery to create a pathway to achieving the 2020 goals. Previous studies have shown that MDA is beneficial for the clearance of inflammation and prevention of subsequent scarring, but we show here that it needs to be coupled with sustainable reductions in transmission (most likely through environmental and water and sanitation improvement) before the elimination of blinding disease due to trachoma can be considered realistically. For medium- and low-endemicity communities at baseline, the model generates far fewer new cases of TT and CO, and a combination of surgery to clear the backlog of TT cases and antibiotics to clear infection may be enough to eliminate blindness. This conclusion is also implied by Table 2, since the aim of a “transmission reduction” campaign is to change permanently the conditions propitious for a highly-endemic setting into those of a low-endemic (or non-endemic) setting. Our model suggests that antibiotics can reduce trachoma in communities such as Kongwa, but a secular trend will be necessary to keep it from returning. Summaries of the potential impact of interventions such as those provided in Table 2, may prove very useful for program managers to optimize their decisions at the beginning or during interventions, particularly in resource-constrained settings, where careful consideration needs to be given to those interventions or combinations thereof which will provide the best value for money. Similar model-based analyses have allowed the systematic exploration of combining existing malaria control interventions (i.e. in the absence of an effective vaccine)^31^ which is now a cornerstone of the debate on malaria elimination. Such summaries of the paths to elimination will need to be accompanied by health-economic analyses in future work to obtain greater clarity of the relative cost-benefits and cost-effectiveness of the strategies, and therefore the overall effort necessary to implement each of the interventions simulated here. In addition to studies conducting scenario analyses, important next steps would be to fit mathematical models to post-intervention longitudinal data, once sufficient data have been collected, as well as extend the complexity of models to allow for population immigration and the inclusion of regions of differing endemicity (as have Lietman and colleagues^10^).

In conclusion, trachoma disease sequelae will continue to develop in the years to come, particularly in highly endemic regions, and so there is a need for the ongoing provision of services, such as surgery for trichiasis, even when the prevalence of active disease is dropping. Evidence for the effectiveness of the F&E intervention is needed to allow transmission reduction to be achieved with greater certainty than is currently possible. The GET2020 program goals may yet be achievable, but unless infection can be eliminated with mass antibiotics, then a substantial and sustained effort will be required to provide surgery and continue implementing all components of the SAFE strategy.

## Supplementary Material

Appendix
