# Supplementary material for: Estimating the Future Impact of a Multi-Pronged Intervention Strategy on Ocular Disease Sequelae Caused by Trachoma: A Modeling Study
Source: Ophthalmic Epidemiol. 2015 Dec 14;22(6):394–402. doi: 10.3109/09286586.2015.1081249 (PMC4841017; doi:10.3109/09286586.2015.1081249)
Supplement: Appendix [file iope_a_1081249_sm6943.doc]

**Appendix**

**Model structure**

The susceptible-infectious model used here channels the population through successive susceptible and infected stages, indexed by the parameter *i*. Each susceptible ($S_{i}$) and infected ($I_{i}$) compartment is connected to the next compartment above it so that individuals pass up a ‘ladder’ of infection. The following partial differential equations describe the flow from one compartment to another in continuous age and time, both of which are discretized for the computer simulation.

$$\frac{\partial S_{i}(a,t)}{\partial t}+\frac{\partial S_{i}(a,t)}{\partial a}=-\beta S_{i}(a,t)\int_{a'} \frac{w(a,a')\sum_{j} \rho_{j}I_{j}(a',t)}{\sum_{j} I_{j}(a',t)+S_{j}(a',t)}da'-\mu(a)S_{i}(a,t)+\upsilon_{i-1}I_{i-1}(a,t)$$

$$\frac{\partial I_{i}(a,t)}{\partial t}+\frac{\partial I_{i}(a,t)}{\partial a}=\beta S_{i}(a,t)\int_{a'} \frac{w(a,a')\sum_{j} \rho_{j}I_{j}(a',t)}{\sum_{j} I_{j}(a',t)+S_{j}(a',t)}da'-\mu(a)I_{i}(a,t)-\upsilon_{i}I_{i}(a,t)$$

Here, $S_{i}$ and $I_{i}$ are functions of age *a* and time *t*;$\beta$ is the transmission parameter from infected to susceptible states; $w(a,a')$ is a mixing matrix (in the discretized version) describing the rate of mixing between individuals of age $a$ and $a'$ $\left(w(a,a')=\varepsilon\delta_{a,a'}+(1-\varepsilon)\frac{N_{a'}}{\sum_{a'} N_{a'}}\right)$, ^1^ where $\delta_{a,a'}$ is the Kronecker Delta;^2^ $N_{a'}$ is the number of individuals of age $a'$and $\varepsilon$is a mixing parameter ranging from 0 (random; each age group mixing equally with every other) to 1 (assortative; each age group mixing only with itself), here set to 0.5; $\rho_{j}$ is the infectivity of infected individuals in compartment $I_{j}$; $\mu(a)$is the death-rate at age *a* obtained from the 2001 World Health Organization (WHO) Tanzanian life table (<http://www3.who.int/whosis/menu.cfm?path=whosis,life>); and $\upsilon_{i}$ is the recovery rate of individuals in compartment $I_{i}$.

The recovery rate per individual $\upsilon_{i}$, from infection *i*, is assumed to follow an exponential function of *i* that starts at a ‘naive’ (no prior infection) rate of $\upsilon_{0}$and saturates at a maximum rate $\upsilon_{\infty}$, $\upsilon_{i}=(\upsilon_{0}-\upsilon_{\infty})e^{-\gamma.i}+\upsilon_{\infty}$

The exponent $\gamma$is the parameter over which this function is fitted to the data of Bailey et al^3^, with results shown in Figure 2a ($\gamma$ represents the rate at which the recovery rate rises with each infection experienced). The value of$\gamma$used in the fitted simulations was 0.7.

We assume the infectivity of an individual $\rho_{i}$ is linearly proportional to their bacterial load $l_{i}$, which we assume is a function of the number of previous infections experienced by that individual, in agreement with the observed decline in load with age in trachoma endemic communities.^4-7^ We chose an exponential function such that the load declines from its initial value $l_{0}$ described by, $l_{i}=l_{0}e^{-\varphi i}$

The parameter $\varphi$ can be estimated from age-stratified data on bacterial load (eg, we fit the function to the data of West et al^7^ in our illustration), and it represents the rate of decline in bacterial load with each infection experienced. The value of $\varphi$ used in the fitted simulations was 0.05. The load, when normalized to lie between 0 and 1, then becomes the infectivity $\rho_{i}$ of the population in compartment *i* .

**Model parameters; effect of interventions on infection; sensitivity analyses**

***Model parameters***

Supplementary Table S1: Parameter values used in the model, with 95% confidence intervals where appropriate.

| **Parameter** | **Parameter definition** | **Maximum likelihood estimate (95% CI)** |
| --- | --- | --- |
| *Transmission dynamics parameters (estimated from model fit)* | | |
| 1/$\upsilon_{0}$ | Mean duration of first infection | 15.1 (6.5-23.3) months |
| 1**/**$\upsilon_{\infty}$ | Mean duration of infection after multiple prior infections | 2.8 (2.4-3.2) months |
| $\gamma$ | Rate of drop in duration of infection per prior infection | 0.7 (0.1-∞) infection^-1^ |
| $l_{1}$ | Infection load per person at first infection | 1.1 (0.9-1.3) x10^5^ copies *Chlamydia trachomatis* omp1 genotype per swab |
| $\varphi$ | Rate of drop in infection load per prior infection | 0.05 (0.03-0.07) infection^-1^ |
| $\beta$ | Transmission coefficient: rate of transmission (per year) of infection between individuals | 27.7 (21.8-35.1) year^-1^ |
| *Disease progression parameters (estimated from model fit)* | | |
|  | Threshold number of infections prior to TS | 102 infections |
|  | Threshold number of infections prior to TT | 151 (119-170) infections [plausible bounds] |
|  | Rate of progression to CO without further infection | 0.033 (0.02-0.055) year^-1^  [plausible bounds] |
| *Treatment-related parameters (from literature)* | | |
|  | Efficacy of single-dose azithromycin treatment | 95% |
|  | Population coverage of MDA | 86% |

CI, confidence interval; CO, corneal opacity; MDA, mass drug administration; TS, trachomatous scarring; TT, trachomatous trichiasis

***Effect of mass drug administration (MDA) on infection***

Using the transmission dynamics parameters from the hyperendemic setting, we simulated the administration of multiple antibiotic treatment rounds (contrasting the effect of 3 vs 10 annual rounds to populations whose level of transmission was set to mimic hyperendemicity (overall infection prevalence >20%); mesoendemicity (10–20%), and hypoendemicity (<10%)^8^. Different endemic levels can be generated by adjusting the value of the transmission coefficient (the $\beta$ parameter). We examined the return of infection through time after cessation of MDA for the 3 endemicity levels described above.

***Sensitivity analyses for the Global Elimination of blinding Trachoma by 2020 (GET2020) program goals***

Sensitivity analyses were performed to determine plausible outcomes for the quantities relating to the GET2020 program goals, namely the time taken to achieve a 1/1,000 prevalence of trachomatous trichiasis (TT) and the treatment outcome for the incidence of corneal opacities (CO). In each case, upper and lowermost limits of the 95% confidence intervals for the transmission rate ($\beta$) and duration of infection (1/$\upsilon_{1}$, 1/$\upsilon_{\infty}$) parameters were taken in combinations that led to the quickest and slowest possible rates of progress along the ladder of infection (these 2 combinations were: high $\beta$, low $\upsilon$ and low $\beta$, high $\upsilon$). Given these new rates of progress along the ladder of infection, the threshold for TT required to reproduce the age-prevalence values seen in the data would also differ, we thus recalculated these thresholds before determining the effect of treatment. The recalculated threshold values for the development of TT were 119 and 170 at the lower and upper end, respectively (151 for the best-fitting parameters). We used these lower and upper thresholds for TT as ‘plausible’ values (as outlined in the context of the HIV pandemic^9-10^) consistent with the data, yet acknowledging that the hyperendemic setting whose data we used to fit the model and obtain the TT threshold, may not have been at equilibrium, or that the baseline infection prevalence may have been overestimated^11^ in which case the TT threshold value estimated may be too low or high. The resulting upper and lower plausible values for the time taken to achieve the TT-based GET2020 program goal are given in the main text.

We also examined the effect of treatment on the population progress toward CO, using plausible upper and lower values for the rate of progress between TT and CO, obtained by altering this rate of progress while maintaining a good visual fit to the CO prevalence data at baseline (see Table 1). The overall incidence trajectories for the upper and lower plausibility bounds are essentially the same in all 3 cases, aside from an offset in the initial values and the size of the fall in CO incidence, due to the different rates of progress toward CO in the upper and lower cases. A long-term effect of treatment on CO incidence is also observed in each case, with annual incidence taking almost 80 years to return to its pre-treatment level, but failing to bring the incidence down to the GET2020 program prescribed level of 1/10,000 persons.

**References**

1. Anderson, R. and R. May (1992). Infectious diseases of humans: dynamics and control, OUP.

2. Boas, M. L. (1983). Mathematical methods in the physical sciences. New York, John Wiley & Sons.

3. Bailey, R., T. Duong, R. Carpenter, H. Whittle and D. Mabey (1999). "The duration of human ocular *Chlamydia trachomatis* infection is age dependent." Epidemiol Infect **123**(3): 479-486.

4. Solomon, A. W., M. J. Holland, M. J. Burton, S. K. West, N. D. Alexander, A. Aguirre, P. A. Massae, H. Mkocha, B. Munoz, G. J. Johnson, R. W. Peeling, R. L. Bailey, A. Foster and D. C. Mabey (2003). "Strategies for control of trachoma: observational study with quantitative PCR." Lancet **362**(9379): 198-204.

5. Solomon, A. W., M. J. Holland, N. D. Alexander, P. A. Massae, A. Aguirre, A. Natividad-Sancho, S. Molina, S. Safari, J. F. Shao, P. Courtright, R. W. Peeling, S. K. West, R. L. Bailey, A. Foster and D. C. Mabey (2004). "Mass treatment with single-dose azithromycin for trachoma." N Engl J Med **351**(19): 1962-1971.

6. Burton, M. J., M. J. Holland, P. Makalo, E. A. Aryee, N. D. Alexander, A. Sillah, H. Faal, S. K. West, A. Foster, G. J. Johnson, D. C. Mabey and R. L. Bailey (2005). "Re-emergence of *Chlamydia trachomatis* infection after mass antibiotic treatment of a trachoma-endemic Gambian community: a longitudinal study." Lancet **365**(9467): 1321-1328.

7. West, E. S., B. Munoz, H. Mkocha, M. J. Holland, A. Aguirre, A. W. Solomon, R. Bailey, A. Foster, D. Mabey and S. K. West (2005). "Mass treatment and the effect on the load of *Chlamydia trachomatis* infection in a trachoma-hyperendemic community." Invest Ophthalmol Vis Sci **46**(1): 83-87.

8. Lietman, T., T. Porco, C. Dawson and S. Blower (1999). "Global elimination of trachoma: how frequently should we administer mass chemotherapy?" Nat Med **5**(5): 572-576.

9. Grassly, N. C., M. Morgan, N. Walker, G. Garnett, K. A. Stanecki, J. Stover, T. Brown and P. D. Ghys (2004). "Uncertainty in estimates of HIV/AIDS: the estimation and application of plausibility bounds." Sex Transm Infect **80 Suppl 1**: i31-38.

10. Walker, N., N. C. Grassly, G. P. Garnett, K. A. Stanecki and P. D. Ghys (2004). "Estimating the global burden of HIV/AIDS: what do we really know about the HIV pandemic?" Lancet **363**(9427): 2180-2185.

11. Gambhir, M., M. G. Basanez, M. J. Burton, A. W. Solomon, R. L. Bailey, M. J. Holland, I. M. Blake, C. A. Donnelly, I. Jabr, D. C. Mabey and N. C. Grassly (2009). "The development of an age-structured model for trachoma transmission dynamics, pathogenesis and control." PLoS Negl Trop Dis **3**(6): e462.
